# A facile synthesis of K_3_PMo_12_O_40_/WO_3_ crystals for effective sonocatalytic performance

**DOI:** 10.1039/d3ra02531d

**Published:** 2023-05-26

**Authors:** Linjing Li, Feng Li, Taohai Li, Wei Cao

**Affiliations:** a College of Chemistry, Key Lab of Environment Friendly Chemistry and Application in Ministry of Education, Xiangtan University Xiangtan 411105 China; b Nano and Molecular Materials Research Unit, Faculty of Science, University of Oulu P.O. Box 3000 FIN-90014 Oulu Finland hnlth@xtu.edu.cn Wei.Cao@oulu.fi

## Abstract

Proper treatment of hazardous contaminants in the air, land, and water is crucial to environmental remediation. Sonocatalysis, by using ultrasound and suitable catalysts, has shown its potential in organic pollutant removal. In this work, K_3_PMo_12_O_40_/WO_3_ sonocatalysts were fabricated *via* a facile solution method at room temperature. Techniques such as powder X-ray diffraction, scanning electron microscopy (SEM), transmission electron microscopy, and X-ray photoelectron spectroscopy were used to characterize the structure and morphology of the products. By using the K_3_PMo_12_O_40_/WO_3_ sonocatalyst, an ultrasound-assisted advanced oxidation process has been developed for the catalytic degradation of methyl orange and acid red 88. Almost all dyes were degraded within 120 min of ultrasound baths, proving that the K_3_PMo_12_O_40_/WO_3_ sonocatalyst has the advantage of speeding up the decomposition of contaminants. The impacts of key parameters, including catalyst dosage, dye concentration, dye pH, and ultrasonic power were evaluated to understand and reach optimized conditions in sonocatalysis. The remarkable performance of K_3_PMo_12_O_40_/WO_3_ in the sonocatalytic degradation of pollutants provides a new strategy for the application of K_3_PMo_12_O_40_ in sonocatalysis.

## Abstract 2

The K_3_PMo_12_O_40_/WO_3_ composite was made straightforwardly by stirring at room temperature, and the putative photocatalytic mechanism was examined in sonocatalytic degradations of methyl orange and acid red 88 under various circumstances.

## Graphical abstract

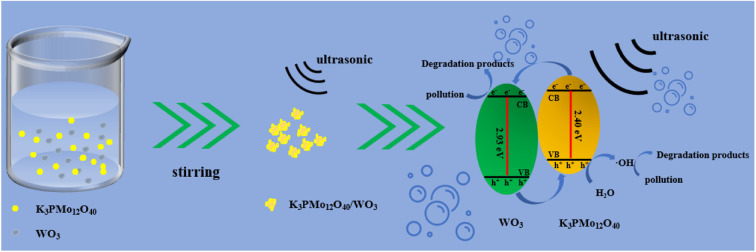

## 1. Introduction

Rapid industrialization is unfortunately accompanied with environmental pollution. Among the various pollutants, organic ones pose relatively serious problems.^1,2^ Therefore, searching for solutions to detoxify organic pollutants has became an urgent issue in many research fields. In this regard, advanced oxidation processes (AOPs), which include ultrasonic decomposition, ozone oxidation, the Fenton reaction, photocatalysis, and other advanced oxidation technologies, have gained increasing interest.^3–5^ For example, the Ce_4_O_7_-modified Bi_4_MoO_9_ (C-BMO) photo-Fenton heterojunction was successfully constructed by Yang *et al.* The degradation of tetracycline (TC) process was improved during the photo-Fenton process, reaching an improved efficiency of 7.69 and 12.28 times higher than those of pure Ce_4_O_7_ and Bi_4_MoO_9_, respectively.^6^ Bi_2_MoO_6_ with exposed {0 1 0} facet has a rich layered structure, and its interaction with Fe-POM with unique electron-trap characteristics can effectively improve charge transfer at the interface. Therefore, Yang *et al.* prepared an efficient and stable heterogeneous photo-Fenton catalyst Fe-POM/Bi_2_MoO_6_.^7^ Under the synergistic action of Fenton reaction and photocatalytic process, CeO_2−*x*_/Bi_2_MoO_6_ heterojunction exhibits outstanding photo-Fenton activity in the degradation of ofloxacin, norfloxacin, and ciprofloxacin antibiotics.^3^ The sonocatalysis process is based on the acoustic cavitation effect, in which ultrasonic vibrations cause bubbles to form, grow, and collapse in solution.^8,9^ The high pressure (≥1000 atm) and high temperature (≥5000 K) are generated at the moment of bubble collapse, and the higher energy generated will cause the dissociation of water to produce hydroxyl radicals (·OH) which react with pollutants to achieve the purpose of destruction.^10,11^ However, degradation using ultrasound alone requires more time and consumes more energy. Therefore, it is urgent to explore and develop efficient sonocatalysts.^12^

As an excellent semiconductor catalyst, tungsten trioxide (WO_3_) has become a material of significant interest in several fields. WO_3_ is a popular material for pollutant removal because of its excellent chemical and physical properties, narrow band gap (2.4–2.8 eV),^13^ good ambient stability, cost-effectiveness, and high recovery rate. WO_3_ was also endowed with considerable attentions in the sonocatalytic removal of pollutants.^14^ According to Li *et al.*, rhodamine B (RhB) degradation with the addition of WO_3_ reached up to 57.9% under ideal sonocatalytic conditions, which is approximately three times higher than that without the addition of catalysts. WO_3_ can remove 68% of methamphetamine under ultrasonic irradiation through the coaction with Er^3+^:Y_3_Al_5_O_12_/KNbO_3_.^15^ However, the formation of superoxide radicals with high recombination rates of electron and hole pairs has been a problem for WO_3_, yet requiring additional treatment of WO_3_.^16^

In recent years, polyoxometalates (POMs) have undergone further research and are now widely applied in several applications.^17^ This is mainly attributed to the reversible redox properties of POMs, which allow the structure of the substance to remain unchanged after participating in the reaction, and the excellent photocatalytic activity that allows POMs to construct effective photocatalytic systems.^18,19^ However, most of the acidic forms of POMs are homogeneous and high soluble in water. This property not only increases the difficulty of separation and recovery but also leads to secondary contamination.^20^ On the other hand, the rapid recombination of electron–hole pairs in POMs caused a low photocatalytic efficiency.^21^ These drawbacks limit the practical application of POMs. It has been found that POMs have low aqueous solubility when constituted of Cs^+^, K^+^, (NH_4_)^+^, or Rb^+^ ions, which greatly improves the recyclability and reduces the cost of POMs.^20^ A variety of studies have shown that combining two catalysts by doping, loading, or constructing heterojunction systems can effectively improve a variety of properties.^22,23^ Therefore, combining WO_3_ and K_3_PMo_12_O_40_ semiconductor materials might reduce the electron–hole pair recombination rate by forming an internal electric field. It can be predicted that the combination of both WO_3_ and K_3_PMo_12_O_40_ can form an efficient catalyst and further promote the degradation of pollutants.

Until now, there is still a paucity of studies related to the sonocatalytic properties of POMs. Herein, we prepared K_3_PMo_12_O_40_/WO_3_ (KW) sonocatalysts *via* a facile solution method at room temperature. Various characterization techniques were used to investigate the structural, morphological, and sonocatalytic properties of KW, to comparatively study the sonocatalytic degradation of the organic dyes methyl orange (MO) and acid red 88 (AR88) by WO_3_, K_3_PMo_12_O_40_ (KPM) and KW, and to further propose possible sonocatalytic degradation mechanisms.

## 2. Experimental

### 2.1. Material and characterization

Sodium tungstate dihydrate (Na_2_WO_4_·H_2_O), potassium chloride (KCl), and phosphomolybdic acid hydrate (H_3_P(Mo_3_O_10_)_4_·*x*H_2_O) were purchased from Sinopharm Chemical Reagent Co., Ltd. Oxalic acid dihydrate (H_2_C_2_O_4_·2H_2_O) was obtained from Foshan Silong Chemical company. Methyl orange (MO, C_14_H_14_N_3_NaO_3_S) and acid red 88 (AR88, C_20_H_13_N_2_NaO_4_S) were purchased from Xiangzhong Chemical Reagent Co. All the chemicals used were of analytical grade and without any further purification.

The crystalline properties of the samples were explored by X-ray diffraction (XRD) patterns (D8 ADVANCE, USA). Morphological characteristics were obtained by scanning electron microscopy (SEM) (JSM-6610LV JEOL, Japan) and transmission electron microscopy (TEM) (JEM, USA). X-ray photoelectron spectroscopy (XPS) was conducted for determining the elementary composition and the surface states on an X-ray photoelectron spectroscopy instrument (ESCALAB 250Xi, USA). The UV-visible diffuse reflectance spectrum (DRS) data of the samples were obtained by UV-visible spectrophotometer (UV-2550, Japan) analysis. Monitoring of ·OH generated during ultrasound using photoluminescence (F-4600, Hitachi, Japan).

### 2.2. Preparation of sonocatalysts

The WO_3_ nanoparticles were synthesized under hydrothermal conditions. Experimental details were as follows: weight 12.5 mmol Na_2_WO_4_·2H_2_O and dissolved into 25 mL of deionized water under magnetic stirring until solid particles completely dissolved. And 3 M HCl was then added dropwise till the pH of the solution became 2. Then, 35 mmol H_2_C_2_O_4_ was added to the mixed solution with continuous stirring until the solid particles were thoroughly precipitated. Deionized water was added to reach a solution volume of 125 mL and obtain WO_3_ sol. Next, 6.25 g of KCl was added to the above solution and stirred to dissolve it. The suspension was then transferred into a Teflon-lined stainless autoclave and the hydrothermal reaction was carried out at 180 °C for 6 h. WO_3_ was collected, and washed with deionized water and ethanol several times. Finally, the solid sample dried in air at 70 °C for 24 h.

K_3_PMo_12_O_40_ was specifically prepared using the following steps. Dispersed 3 g of H_3_PMo_12_O_40_ (dry in a drying oven at 150 °C for 12 h before use to remove moisture molecules) in 20 mL of deionized water. The above solution was stirred for 10 min at room temperature until dissolved thoroughly. With continuous stirring, a solution containing a sufficient amount of KCl (0.184 g dissolved in 10 mL of water) was added dropwise to the above solution at room temperature. The result mixture was stirred for 15 min and left for 24 h. The precipitate was collected, washed several times with deionized water and ethanol, and then dried in air at 70 °C for 24 h.

The following process was used to prepare the sonocatalyst KW. The KPM sample (1 g) was dissolved in 40 mL deionized water and 0.2 g WO_3_ was dissolved in 10 mL H_2_O respectively. Then the WO_3_ solution was added to the KPM solution dropwise gradually. The mixture was stirred at room temperature for 2 h. After mixing finished, the precipitate was collected, washed several times with deionized water and ethanol, and dried in air at 70 °C for 24 h. Because the KPM dispersed in water is in a relatively viscous state, it can be successfully adsorbed on the solid surface of KPM by simply stirring after adding WO_3_.

### 2.3. Exploration of sonocatalysis process

Degrading MO and AR88 as pollutants in an aqueous solution were used to investigate the sonocatalytic activity of the KW. The KW sample was dispersed into 50 mL of dye solution to determine the sonocatalytic activity and the adsorption capacity of the product in the dark. Before using ultrasonic energy, the suspension was magnetically stirred in the dark for 30 min to reach the adsorption–desorption equilibrium of the dye solution on the sonocatalyst. Then, under ultrasonic irradiation, 3 mL of the reaction solution was taken from each mixture at 30 min intervals and centrifuged at 8500 rpm for 10 min to remove the catalyst powder. The ultrasound irradiation lasted 120 min. Different process factors were examined, including sample dosage, initial dye solution concentration, pH, and incident ultrasound power. The final concentration of dyes was analyzed by using a UV-vis spectrophotometer (CARY 600, Agilent, USA) at a wavelength coverage of 200–800 nm. The conversion was calculated by (*C*_0_–*C*)/*C*_0_, where *C* is the concentration of the reactant after irradiation, and *C*_0_ is the initial concentration of the organic pollution.

### 2.4. Hydroxyl radical analysis

The production of ·OH radical is usually demonstrated by a PL method. The hydroxylated compounds are created by oxidizing or disproportionating the hydroxy cyclohexadienyl radicals that are produced when hydroxyl radicals are added to coumarin. As a result, by observing the PL intensity of the reacting solution, we can gather information on the ·OH radicals. 1 mmol of coumarin was dissolved in 1 L of deionized water to create a coumarin solution. The catalyst (50 mg) was added to 50 mL of the coumarin solution. After magnetic stirring in the dark for 30 min, the mixed solutions were ultrasonically irradiated under the same ultrasonic experimental conditions. The reacted solution (3 mL) was removed at the specified reaction time interval, and the catalyst was then removed by centrifuging the mixture for 10 minutes. After centrifugation, the upper clear solution was taken for PL measurement by fluorescence spectrophotometer with an excitation wavelength of 332 nm.

## 3. Results and discussion

### 3.1. X-ray diffraction study

**Fig. 1:**
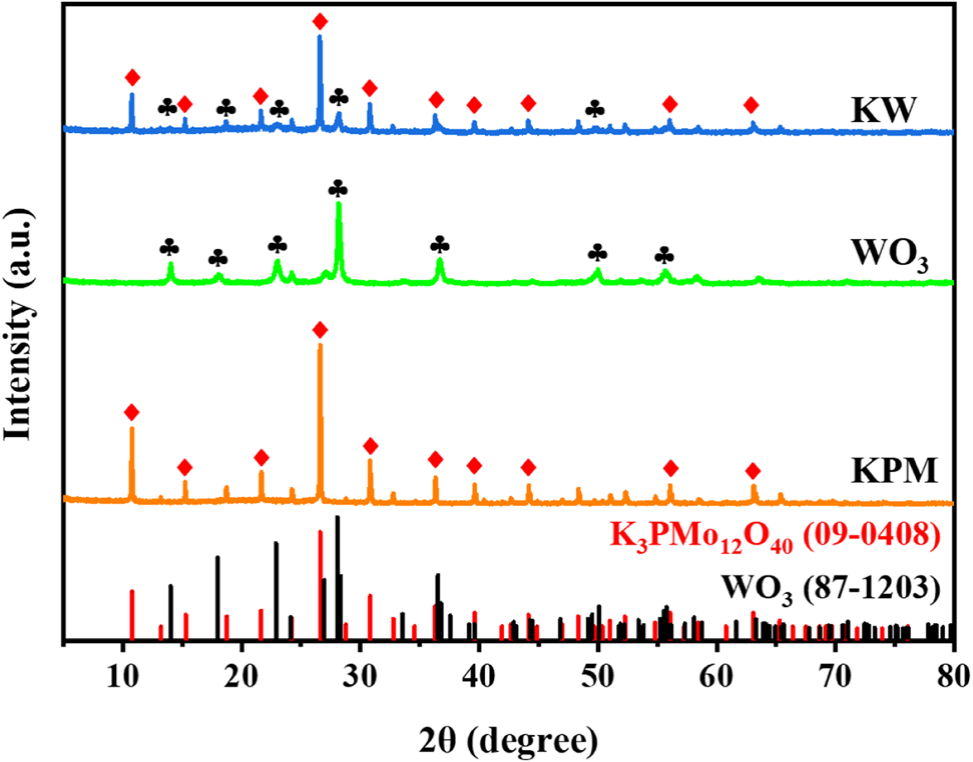
XRD pattern of WO_3_, KPM, and KW samples.

The XRD patterns of WO_3_, KPM, and KW were presented in Fig. 1. The peaks at 2*θ* = 10.7°, 15.2°, 21.6°, 26.6°, 30.8°, 36.2°, 39.5°, 44.1°, 56.0°, and 63.0° were indexed to K_3_PMo_12_O_40_ (JCPDS no. 09-0408), corresponding to the (110), (200), (220), (222), (400), (332), (510), (440), (550), and (651) crystal planes of K_3_PMo_12_O_40_, respectively. The diffraction peaks of WO_3_ (JCPDS no. 87-1203) observed at 2*θ* = 14.1°, 18.0°, 22.9°, 28.1°, 36.6°, 50.1°, and 55.8°, denoting diffractions from the (020), (111), (002), (220), (222), (260), and (044) crystalline surfaces, respectively. From the XRD analysis of sample KW, the peak intensities of KPM were slightly weakened due to the addition of WO_3_. However, the correlation peaks of the two substances can still be seen in the test results, indicating that the combination of the materials would not affect the crystallinity of KPM.

### 3.2. SEM and TEM analysis

**Fig. 2:**
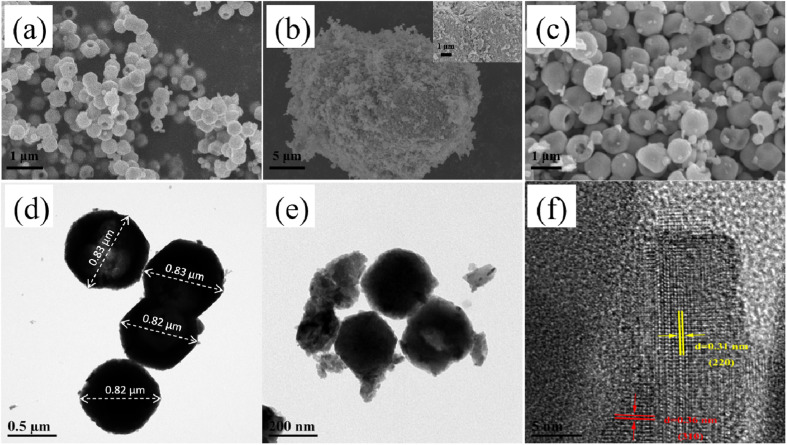
SEM images of (a) KPM, (b) WO_3_, and (c) KW; TEM images of the (d) KPM, and (e) KW samples; HRTEM image of the (f) KW.

Fig. 2(a)–(c) display the SEM images of the KPM, WO_3_, and KW samples, respectively. The SEM image of the KPM particles in Fig. 2(a) demonstrates that nanomaterials are a type of microspheres with smooth surfaces, showing a hollow center and varying particle diameters. As illustrated by the SEM image in Fig. 2(b), the WO_3_ nanoparticles are blocks with irregular shapes. According to the SEM images in Fig. 2(c), WO_3_ particles anchored on KPM and formed KW heterostructures. It is obvious that the surface of the KPM becomes uneven or somewhat defective after attached with the WO_3_. TEM was used to further investigate the microstructure of KPM powder, WO_3_ powder, and WO_3_-doped KPM nanocomposite. According to Fig. 2(d), the KPM samples were depicted as microspheres and particle sizes about 0.82–0.83 μm, which was in good agreement with the SEM results of Fig. 2(a). The TEM image of KW was shown in Fig. 2(e). The image reveals two different types of nanoparticles with various sizes and shapes. The more erratic-shaped particles (referred to as WO_3_ nanoparticles) are attached to the hollow-shaped KPM nanoparticles. The TEM observation demonstrates the formation of KW heterogeneous structures, consistent with the conclusion obtained through SEM. Two distinct forms of well-defined lattice stripes turn out in an HRTEM picture in Fig. 2(f). The spacing of one set of the fringes was *ca.* 0.310 nm, corresponding to the (220) plane of WO_3_, and another one was *ca.* 0.360 nm, corresponding to the (310) lattice spacing of KPM. Therefore, the as-obtained KW heterostructure was composed of KPM and WO_3_.

### 3.3. XPS analysis

**Fig. 3:**
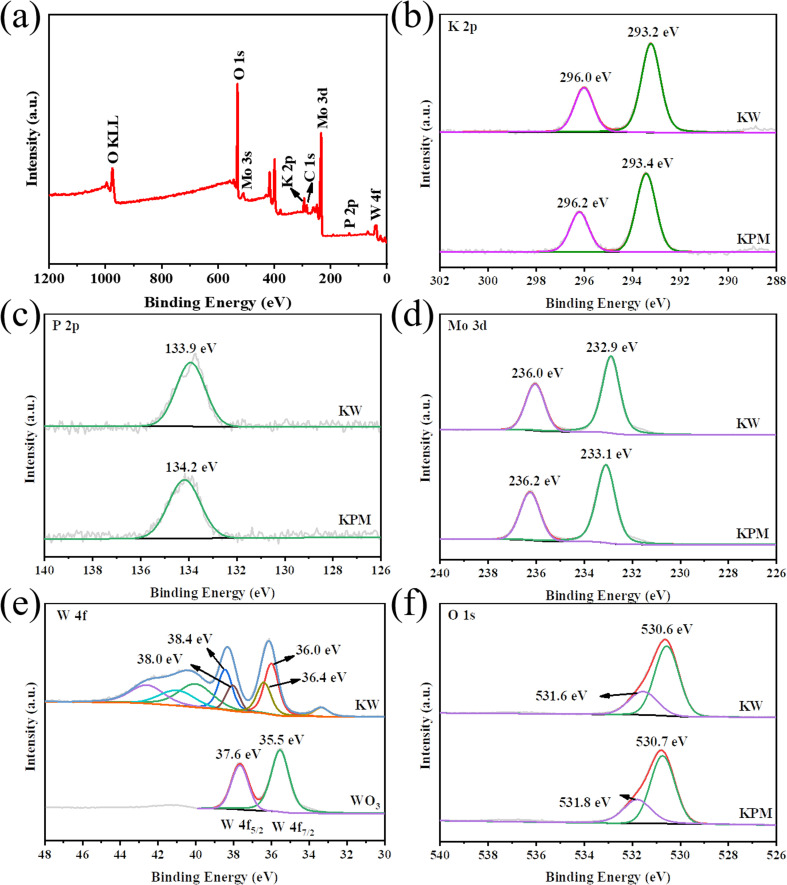
XPS spectra of the KW sample: (a) survey, (b) K 2p, (c) P 2p, (d) Mo 3d, (e) W 4f, and (f) O 1s.

Fig. 3 shows the XPS analysis, which reflects the surface composition and chemical state of KPM modified by WO_3_. The coexistence of K, P, Mo, O, and W elements in the composites was detected by XPS survey spectra (Fig. 3(a)), which confirmed the elemental composition of KW. The binding energy (BE) in the XPS study was calibrated by using the adventurous C 1s peak at 284.6 eV as the reference.^24^ The two prominent peaks in the K 2p XPS spectrum of KPM in Fig. 3(b) at 296.2 eV and 293.4 eV, attributed to K 2p_1/2_ and K 2p_3/2_, respectively, indicate that the K element is in the K^+^ chemical state. The BE of K 2p slightly shifted to lower energies after compounding WO_3_ with KPM, suggesting that the addition of WO_3_ may have changed the chemical environment of K. This effect can also be observed in the XPS spectra of P 2p, Mo 3d, and O 1s. Fig. 3(c) depicts the typical XPS peak of P 2p corresponding to a binding energy peak of 134.2 eV, revealing that the valence state of P in this composite is +5 and the BE of KW was reduced by 0.3 eV compared to that of KPM. In the Mo 3d XPS spectra of both samples, the XPS peaks of Mo 3d_3/2_ and Mo 3d_5/2_ for the KPM sample (Fig. 3(d)) correspond to unique peaks with binding energies of 236.0 eV and 232.9 eV, respectively, which are attributed to Mo(vi) in the Keggin-type heteropolyacid anion [PMo_12_O_40_]_3_^−^.^25^ The change in BE of Mo 3d after WO_3_ modification of KPM further illustrated the change in the chemical environment of KPM. The W 4f spectrum of WO_3_ has two peaks, W 4f_7/2_ (35.5 eV) and W 4f_5/2_ (37.6 eV), and these binding energies show the +6 chemical state of W (Fig. 3(e)).^26^ The W 4f of the KW sample was in a variety of chemical environments, with a slight shift of the BE of the major W^6+^ toward the higher direction. In the different O 1s chemical environments of KPM and KW in Fig. 3(f), the peak of 530.7 eV in KPM corresponded to the O 1s binding energy attributed to the (Mo–O–Mo) bond in [PMo_12_O_40_]_3_^−^.^25^ The other peak at 531.8 eV belongs to the surface OH. The addition of WO_3_ reduces both BEs by 0.1 eV at the same time. The slight shift of the lattice oxygen confirms the decrease in the electron density of KPM due to the addition of WO_3_.^27^

### 3.4. DRS analysis of KW sample

**Fig. 4:**
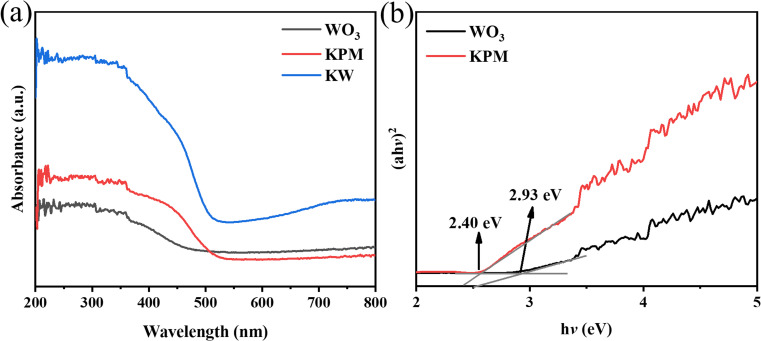
(a) DRS spectrum of KW; (b) plot of (*α*ℏ*ν*)^2^–ℏ*ν* for WO_3_ and KPM samples.

The DRS (in the range of 250–800 nm), band gaps of the WO_3_, KPM, and KW sonocatalysts are shown in Fig. 4(a) and (b). In Fig. 4(a), the absorption peak of pure WO_3_ appears at 450–500 nm. Meanwhile, the absorption value of pure KPM is between 500 and 550 nm. Compared to pure KPM, the KW composite obtained after loading with WO_3_ owns a more pronounced absorption feature at the range of 500–550 nm. Combining the optical absorption edge cases of pure WO_3_ with KPM discussed before, and according to the Kubelka–Munk formula, the corresponding band gap energy (*E*_g_) can be calculated through^28^

1 *α*ℏ*ν* = *A*(ℏ*ν* − *E*_g_)^*n*^

In the above equation (eqn (1)), *α*, ℏ, *ν*, and *A* represent the absorption coefficient, Planck constant, optical frequency, and constant, respectively. According to the ref. 13, WO_3_ and KPM are both direct bandgap semiconductors, thus, *n* is defined to 1/2. Plotting the (*a*ℏ*ν*)^2^–ℏ*ν* relationship, the *E*_g_ of WO_3_ and KPM can be calculated to 2.40 eV and 2.93 eV, respectively (Fig. 4(b)).

### 3.5. Sonocatalytic test results

MO and AR88 were selected as the model of organic pollutants to evaluate the sonocatalytic performance of KW nanocomposite. The sonocatalytic activity of KW nanocomposites was examined by various experiments under various sonication conditions. The sample dosages were tuned to 12.5 mg, 25 mg, 50 mg, and 70 mg, dye concentrations to 5 mg L^−1^, 10 mg L^−1^, 15 mg L^−1^, and 20 mg L^−1^, pH values set to 3, 7, 11, and sonication powers to 120 W, 168 W, and 240 W. By doing so, the ideal circumstances for the cyclic experimental investigation were chosen from them after assessing the degradation rates of each variable on the dyes.

#### 3.5.1. Performance of different catalytic components

**Fig. 5:**
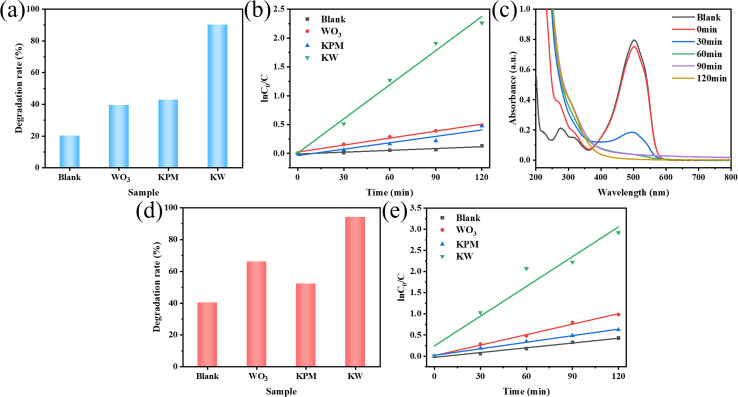
Sonocatalytic degradation comparison of (a) MO and (d) AR88 in the presence of different components; (c) the UV-vis absorption spectrum of the sample KW for MO degradation; kinetics of sonocatalytic degradation of (b) MO and (e) AR88 with different components (experimental conditions: sonocatalyst dosage = 50 mg, initial concentration = 10 mg L^−1^, solution volume = 50 mL, ultrasonic power = 240 W, pH = 7).

Controlled experiments by using only sonication and sonication with component compound in the KW were performed to compare the catalytic activities of these with KW. As shown in Fig. 5(a), the degradation rate of MO was only 19.98% in the state without catalyst. When the KW catalyst was added, the degradation rate increased to 90.13%, which is about 4.5 times higher than the original one. The degradation rates of the composite KW were all higher than these of the individual components. The UV-vis absorption curve of sample KW degraded MO pollution is depicted in Fig. 5(c). It is discovered that the characteristic absorption peak at 502 nm gradually decreases with the ultrasonic irradiation time. The absence of new peaks in the spectra confirms the massive mineralization of MO molecules during this process.

The results of the degradation of AR88 in the presence of different catalysts are presented in Fig. 5(d). The degradation efficiency of the dye AR88 in the absence of the catalyst is 40.26%, while the degradation efficiency of WO_3_, KPM, and KW catalysts are 66.13%, 52.23%, and 94.71%, respectively. It is obvious that the composite catalyst KW has the best catalytic activity. We use first-order kinetic equations to fit the data to better quantify and explain the ultrasonic catalytic degradation of materials. The kinetic fitting results in Fig. 5(b) and (e) demonstrate that the KW composite has a higher reaction rate constant than the other materials'. A possible explanation for this result is proposed. The formation of heterojunction reduces the fast electron–hole recombination rates in both catalysts. On the other hand, the composite catalyst KW combines the advantages of both individual catalysts and further improves the degradation efficiency.

#### 3.5.2. Effect of sonocatalyst dosage

**Fig. 6:**
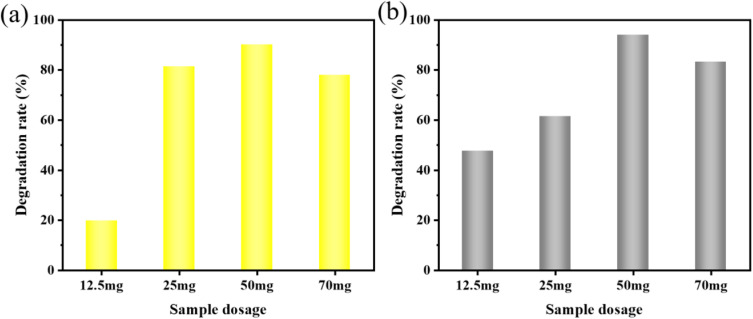
Effect of different KW sonocatalyst dosages on the sonocatalytic degradation of (a) MO and (b) AR88 (experimental conditions: initial concentration = 10 mg L^−1^, solution volume = 50 mL, ultrasonic power = 240 W, pH = 7).

The optimal amount of catalyst is critical for the effective sonocatalyzed degradation of dye contaminants. Depending on the set degradation experimental conditions, the variation of dye color removal rate with time was obtained, as shown in Fig. 6(a). Within a certain range of sample dosages, the degradation rate was proportional to the sample dosages. The sonocatalytic degradation rates of MO were 15.8% (12.5 mg), 81.9% (25 mg), 90.5% (50 mg), and 77.7% (70 mg) when we added different dosages of the samples, respectively. The information demonstrated that a sample quantity of 50 mg produced the best sonocatalyst degradation results. To carry out the studies, 50 mg was determined to be the ideal dosage. However, the degradation effect of 70 mg was lower than that of 50 mg. This issue indicated that a large amount of sonocatalysts facilitates the degradation of MO. The increase in the sonocatalytic decolorization of MO with the dosage of KW nanocomposites was attributed to the increase in the available active reaction sites, thus generating more hydroxyl radicals (·OH) for MO degradation.^29^ However, increasing the dosage of KW nanocomposites from 50 mg to 70 mg does not lead to increase of degradation rate. This may be because the excess sonocatalyst in the solution scatters and blocks the ultrasound waves, lowering the energy and the rate of the sonocatalytic degradation reaction close to the surface of the sonocatalyst.^30–33^

To further investigate the applicability of the catalyst, the AR88 dye solution was degraded under the same conditions. According to Fig. 6(b), the rates of degradation for 12.5 mg, 25 mg, 50 mg, and 70 mg after 120 minutes were 47.6%, 61.4%, 94.0%, and 65.3%, respectively. For further studies, a dosage of 50 mg was chosen as the sample amount for subsequent studies on the degradation of AR88.

#### 3.5.3. Effect of initial dye concentration

**Fig. 7:**
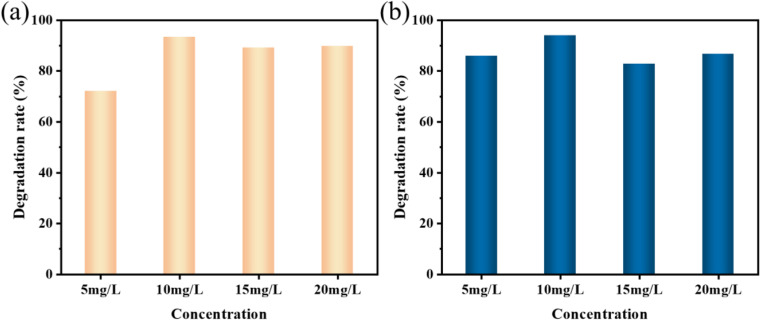
Sonocatalytic degradation efficiency different initial concentrations of (a) MO and (b) AR88 (experimental conditions: sonocatalyst dosage = 50 mg, solution volume = 50 mL, ultrasonic power = 240 W, pH = 7).

From an application point of view, the sonocatalytic degradation rate of dyes is highly dependent on their initial concentration. The volatility of dye concentration in wastewater makes it necessary to further investigate its effect on the ultrasonic degradation effectiveness. By adjusting the initial dye concentration in the range of 5 to 20 mg L^−1^ at a fixed dosage of KW nanocomposite (50 mg) and reaction duration (120 min), the impact of initial MO concentration on the sonocatalytic degradation efficiency (%) was examined. Fig. 7(a) provides the degradation rates of the samples to degrade contaminants of different concentrations. It can be noticed that the degradation rates were 72.6%, 93.4%, 90.1%, and 90.8% when the concentration of the MO dye was between 5 mg L^−1^ and 20 mg L^−1^, respectively. The best degradation effect was achieved when the MO concentration was 10 mg L^−1^. As shown in Fig. 7(b), the degradation rate of AR88 dye at 10 mg L^−1^ was 94% in 120 min. It can be concluded that when either higher or lower dye concentrations are not conducive to KW sonocatalytic degradation of pollutants. This phenomenon could be caused by the fact that MO and AR88 dyes only have a finite number of active sites for degradation. Low dye concentrations lead to poor degradation because the molecules cannot fully attach to the adsorption sites on the catalyst surface. When the dye concentration is too high, the sample molecules gather on the catalyst surface, blocking the dye and catalyst reaction and producing unsatisfactory results. However, at the dye solution concentration of 10 mg L^−1^, the dye molecules cover the active sites on the catalyst surface, allowing the dye molecules to completely react with KW.^29,34,35^

#### 3.5.4. Effect of pH

**Fig. 8:**
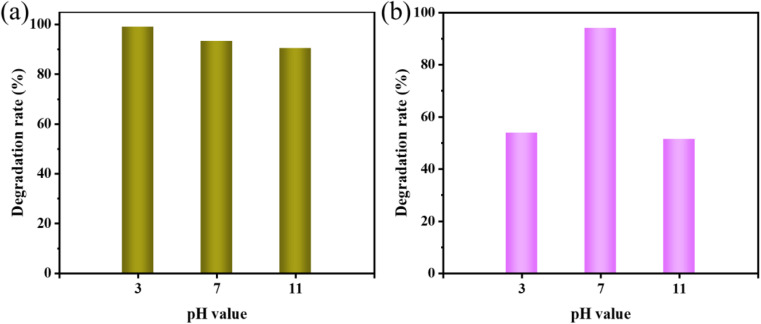
Effect of different pH values on sonocatalytic degradation of (a) MO and (b) AR88 (experimental conditions: sonocatalyst dosage = 50 mg, solution volume = 50 mL, initial concentration = 10 mg L^−1^, ultrasonic power = 240 W).

The pH of the dye also affects how it is degraded in sonocatalysis. Therefore, we investigated the effect by adjusting the dye to different pH values. When studying the effect of pH on dye degradation, other parameters were kept constant. The pH values of the dyes were adjusted to 3, 7, and 11. Fig. 8(a) gives the degradation situation of MO dye solutions at different pH values. After 120 min, the degradation rates were 99%, 93%, and 90.5% for pH = 3, 7, and 11, respectively. From the results, we can realize that the samples showed positive degradation of the pollutants under acidic and neutral conditions, while the degradation of the dye pollutants was not favorable under alkaline conditions. For AR88 dyes, KW had an optimal degradation at pH = 7 (Fig. 8(b)). The aforementioned results might be explained by the fact that when pH changes, electrostatic repulsion between the KW and dye surface rises. This prevents dye molecules from accessing the KW surface, slowing down the pace at which the contaminant degrades.

#### 3.5.5. Effect of ultrasonic power

**Fig. 9:**
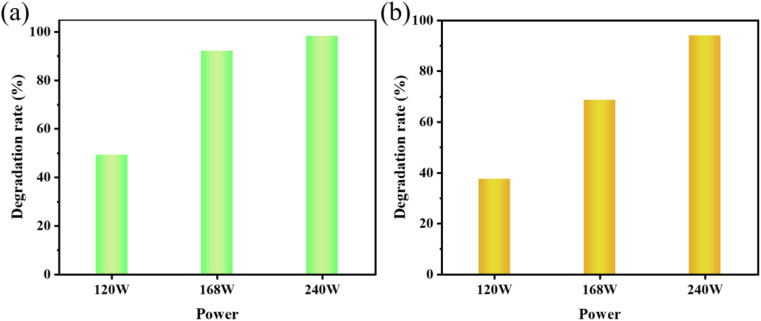
(a) The effect of ultrasonic power on the sonocatalytic degradation of MO (experimental conditions: sonocatalyst dosage = 50 mg, initial concentration = 10 mg L^−1^, pH = 3); (b) the effect of ultrasonic power on the sonocatalytic degradation of AR88 (experimental conditions: sonocatalyst dosage = 50 mg, initial concentration = 10 mg L^−1^, pH = 7).

The impact of parametric ultrasonic power on cavitation processes cannot be disregarded.^36^ Different ultrasonic powers (120 W, 168 W, and 240 W) were employed in the studies to examine the impact of these changes on the degradation of dyes in the presence of sonocatalysts. The results show that the degradation power was 91%, 93%, and 98% at 120 min when the ultrasound power was 120 W, 168 W, and 240 W, respectively (Fig. 9(a)). The states of AR88 degradation by sonocatalyst at 120 W, 168 W, and 240 W are shown in Fig. 9(b). It can be concluded that the degradation rate of AR88 dye by KW was the highest at 240 W. Thus, 240 W was selected as the optimized amount for performing the experiments. The experiments showed that the catalytic efficiency of the samples enhanced with power. The quick collapse of the cavitation bubble caused by the rise in ultrasonic power and increased ultrasonic energy promotes the net production of ·OH radicals at the radical sites.^36,37^ Furthermore, high ultrasonic power causes the solution to become more turbulent, which accelerates the mass transfer of dyes, intermediates, and reactive groups from the native solution to the catalyst surface. The reason for this trend could be that as the ultrasonic power increases, more active bubbles can be produced at the same time, which facilitates the production of more ·OH.^38,39^

#### 3.5.6. Reusability of the sonocatalyst

**Fig. 10:**
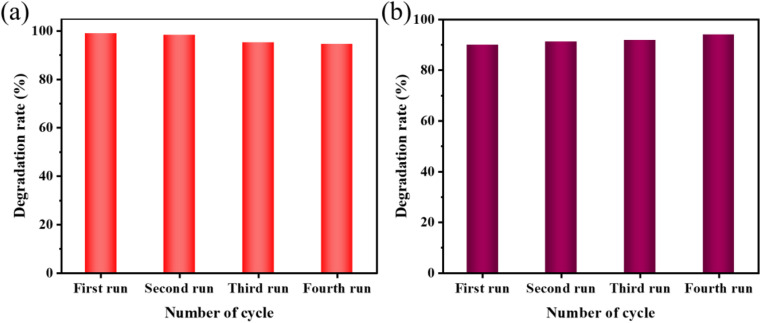
(a) Results of reusability test for the sonocatalytic degradation of MO (experimental conditions: sonocatalyst dosage = 50 mg, initial MO concentration = 50 mg L^−1^, ultrasonic power = 240 W, pH = 3); (b) results of reusability test for the sonocatalytic degradation of AR88 (experimental conditions: sonocatalyst dosage = 50 mg, initial MO concentration = 50 mg L^−1^, ultrasonic power = 240 W, pH = 7).

Consecutive application of the sonocatalyst by maintaining its activity is critical for its usage. Decorating catalysts with suitable dopants has been demonstrated to improve performance and enhance reusability.^40^ The samples were used for four cycles of sonocatalyzed degradation of MO dye and AR88 dye with 50 mg of sample, 10 mg L^−1^ of dye concentration, 10 mL of dye volume, pH 3 for MO dye (pH 7 for AR88), and 240 W of ultrasonic power to further investigate the stability and repeatability of the sonocatalysis. The sonocatalyst was removed from each experiment, cleaned, dried, and then utilized for the subsequent cycle. As seen in Fig. 10(a), the data display a negligible decrease in MO after four repeated cycles. The MO degradation rates after four cycles of KW sonocatalysis were 96%, 98%, 95%, and 94%, respectively. Similarly, the degradation rate of AR88 was maintained at a high level of more than 85% (Fig. 10(b)). According to these findings, KW may be employed as a stable sonocatalyst and is long-lasting and efficient in treating organic dyes.

### 3.6. Possible sonocatalytic degradation mechanism

**Fig. 11:**
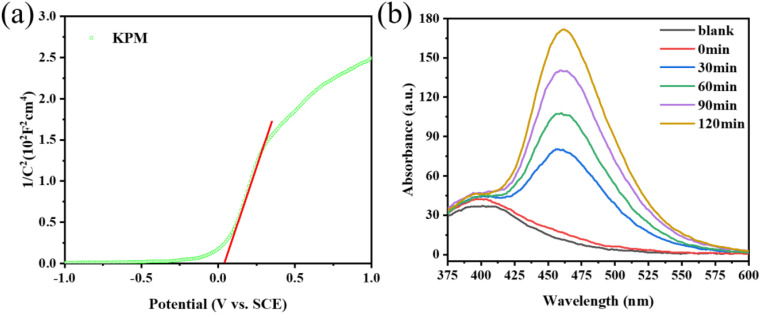
(a) Mott–Schottky plot of KPM; (b) PL spectra of KW.

Previous studies showed that the band structure of the catalyst used for the sonication process is a key factor affecting the catalytic activity. Based on the findings of the DRS spectroscopic band gap measurement, the *E*_g_ of KPM is determined to 2.40 eV. Fig. 11(a) displays the Mott–Schottky measurements of KPM. It can be concluded that the flat-band (*E*_FB_) potential of the KPM is 0.04 eV (0 V *vs.* SCE). Thus, the conduction band (*E*_CB_) edge of KPM is −0.06 eV (compared to NHE). Combined with the above analysis, the valence band (*E*_VB_) of KPM can be calculated as 2.34 eV based on *E*_VB_ = *E*_CB_ + *E*_g_. The band structure of WO_3_ was obtained by referring to the relevant ref. 13.

The production of ·OH radicals may affect activity of acoustic degradation. The reaction of coumarin with hydroxyl radicals produces a variety of hydroxylation products, so coumarin can be used as a fluorescent probe for hydroxyl radicals.^41^ With the addition of KW sonocatalyst and increasing sonication duration, as seen in Fig. 11(b), the PL intensity of coumarin solution progressive increases, which indicates the production of ·OH radicals. As a result, the interaction of a sizable quantity of ·OH in solution with organic dye molecules is responsible for the acoustic degrading activity on dyes of KW. During ultrasonic action, the solution creates a cavitation effect, and the cavitation bubbles produced by this effect can be coupled with adiabatic heating of the bubble vapor phase by speedy decomposition to yield significant local transition temperatures and pressures.^42^ As a result, organic substances (such as MO or AR88) close to the bubble/water contact may experience a thermal breakdown or secondary processes involving MO (or AR88) that result in their mineralization or deterioration.^43^ According to Li *et al.*, the azo bond (–N=N–) of MO is easily broken.^44^ The ·OH radicals generated during the sonocatalysis process are connected to the chromophore, which promotes the breaking of the –N=N– bond after contact with MO, and finally obtains inorganic small molecule such as CO_2_ and H_2_O. In a word, MO undergoes mineralization and resulting in the disappearance of orange.^45–48^

Sonocatalysis has been utilized for decades to degrade and remove organic contaminants from water, which offers excellent technological support for the advancement of environmental friendliness. However, based on a review of the literature, there has not yet been a fairly good explanation of the process in this respect, which is somewhat unfavorable for the advancement of ultrasonics.^15^ Therefore, it is necessary to understand the reaction process of decomposition of MO and AR88 by the action of KW ultrasound. In this study, we provide a potential mechanism to help explain potential fluctuations in KW during the catalytic process.

**Fig. 12:**
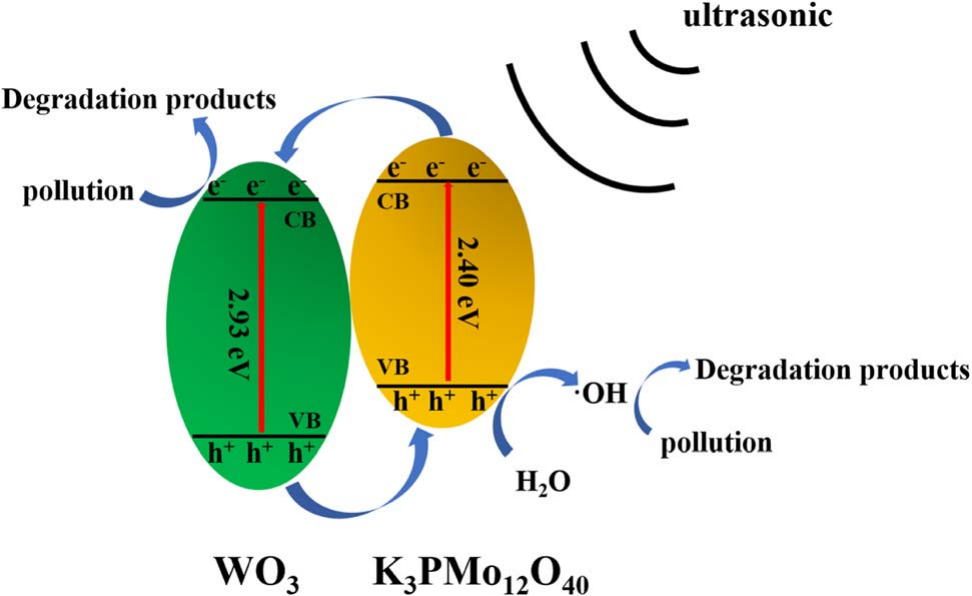
Possible sonocatalytic mechanism.

Here, we refer to the energy band theory to describe the mechanism of sonocatalyzed degradation. It is well known that powerful ultrasonic radiation induces a low-pressure and high-pressure cycling environment in aqueous solutions. Active bubbles are formed in the low-pressure cycle and expand until they collapse when exposed to the high-pressure cycle. A very high pressure is developed during this process, which induces a polarization charge on the surface of the K_3_PMo_12_O_40_/WO_3_ sonocatalyst, generating a hole (h^+^) and an electron (e^−^) at the valence band position (VB) and the conduction band (CB) position of the catalyst, respectively, and an internal electric field. Since the conduction band potential of K_3_PMo_12_O_40_ is more negative than that of WO_3_, the ultrasound-induced e^−^ tends to transfer more to the conduction band of WO_3_. The free-moving e^−^ and h^+^ can be separated rapidly by the electric field. The generated h^+^ can form ·OH by interacting with H_2_O, which in turn interacts with organic pollutants (MO/AR88) when in contact with them, leading to catalytic degradation. On the other hand, ultrasound causes e^−^ to gain enough energy to transfer to the CB of the catalyst and then to the surface of the catalyst as free-moving electrons, enabling them to react with heteroatoms or molecules. The specific process is as follows (Fig. 12).

A few researchers have investigated the degradation of organic dyes using ultrasound techniques (Table 1). To show the benefits of K_3_PMo_12_O_40_/WO_3_ composites in the sonocatalytic degradation of organic dyes, the sonocatalytic activity of K_3_PMo_12_O_40_/WO_3_ was compared with other types of sonocatalysts that have been reported in literature. It can be seen that K_3_PMo_12_O_40_/WO_3_ has more significant sonocatalytic activity in the degradation of organic dyes compared to the published sonocatalysts such as Bi_2_WO_6_, rutile TiO_2_, g-C_3_N_4_, and LuFeO_3_ materials. The results further indicate that K_3_PMo_12_O_40_/WO_3_ is a material with great potential for application in the sonocatalytic degradation of organic pollutants.

**Table 1:** The sonocatalytic activity of K_3_PMo_12_O_40_/WO_3_ compared with some reported catalysts

Catalyst	Pollutant	Concentration (mg L^−1^)	Dosage (g L^−1^)	Time (min)	pH	Degradation (%)	Ref.
Bi_2_WO_6_	MO	10	1.00	60	7	83	He, *et al.*^49^
Rutile TiO_2_	MB	10	0.50	150	3	99	Wang, *et al.*^50^
g-C_3_N_4_	MO	10	1.00	180	7	48	Song, *et al.*^51^
LuFeO_3_	MO	5	4.00	30	—	73	Zhou, *et al.*^52^
K_3_PMo_12_O_40_/WO_3_	MO/AR88	10	1.00	120	3/7	98	This work

## 4. Conclusion

In conclusion, this work showed that KW nanocomposites can be synthesized simply by stirring at room temperature and can be further applied for the sonocatalytic degradation of dyes of MO and AR88. XRD patterns depicted that the synthesized complexes own high crystallinity, and no impurities were found. SEM images and TEM images indicated that WO_3_ forms heterojunctions on the surface loading of KPM, which accelerated the separation of e^−^ and h^+^ and promoted the transfer of electrons, which contributed to the sonocatalytic degradation performance of the composite. XPS spectra revealed that WO_3_ was successfully loaded into the polyoxometalates KPM. The results have demonstrated that the amount of catalyst, the initial concentration of the dye, the pH of the dye solution, and the intensity of the ultrasonic treatment all have varying degrees of effect on the removal of the organic dyes MO and AR88 during the experimental procedure. The degradation of pollutants by the KW sonocatalyst was significantly improved by optimizing these key parameters. According to the experimental results, the selection of optimal experimental conditions after the addition of the KW sonocatalyst could increase the ultrasonically catalyzed degradation rates of MO and AR88 to 96% and 94%, respectively. The reusability test verified the tremendous potential of KW from an application standpoint in multiple runs without significant reduction in sonocatalytic activity. Along with a systematic study of the KW sonocatalyst and its application conditions, this work is hoped to debut a research trend in using polyoxometalates oxides for wastewater remediation through sonocatalysis.

## Conflicts of interest

The authors declare that they have no known competing financial interests or personal relationships that could have appeared to influence the work reported in this paper.

## Supplementary Material
